# Automated generation of node‐splitting models for assessment of inconsistency in network meta‐analysis

**DOI:** 10.1002/jrsm.1167

**Published:** 2015-10-13

**Authors:** Gert van Valkenhoef, Sofia Dias, A. E. Ades, Nicky J. Welton

**Affiliations:** ^1^ Department of EpidemiologyUniversity of Groningen, University Medical Center GroningenGroningenThe Netherlands; ^2^ School of Social and Community MedicineUniversity of BristolBristolUK

**Keywords:** network meta‐analysis, mixed treatment comparisons, meta‐analysis, node splitting, model generation, Bayesian modelling

## Abstract

Network meta‐analysis enables the simultaneous synthesis of a network of clinical trials comparing any number of treatments. Potential inconsistencies between estimates of relative treatment effects are an important concern, and several methods to detect inconsistency have been proposed. This paper is concerned with the node‐splitting approach, which is particularly attractive because of its straightforward interpretation, contrasting estimates from both direct and indirect evidence. However, node‐splitting analyses are labour‐intensive because each comparison of interest requires a separate model. It would be advantageous if node‐splitting models could be estimated automatically for all comparisons of interest.

We present an unambiguous decision rule to choose which comparisons to split, and prove that it selects only comparisons in potentially inconsistent loops in the network, and that all potentially inconsistent loops in the network are investigated. Moreover, the decision rule circumvents problems with the parameterisation of multi‐arm trials, ensuring that model generation is trivial in all cases. Thus, our methods eliminate most of the manual work involved in using the node‐splitting approach, enabling the analyst to focus on interpreting the results. © 2015 The Authors Research Synthesis Methods Published by John Wiley & Sons Ltd.

## 1 Introduction

Network meta‐analysis (Caldwell *et al*., 2005; Lumley, 2002; Lu and Ades, 2004) is a general framework for the synthesis of evidence from clinical trials comparing any number of treatments. It includes pair‐wise meta‐analysis (Hedges and Olkin, 1985) and indirect‐comparison meta‐analysis (Bucher *et al*., 1997; Song *et al*., 2003) as special cases (Jansen *et al*., 2011; Dias *et al*., 2013a). The key assumption underlying any meta‐analysis is exchangeability of the included trials (Lu and Ades, 2009). Violations of the exchangeability assumption can manifest as heterogeneity (within‐comparison variability) or inconsistency (between‐comparison variability). Although the most important defense against such violations is the a priori evaluation of trial design and population characteristics, the (statistical) evaluation of both heterogeneity and inconsistency is also important to ensure valid results from a network meta‐analysis.

A number of methods have been proposed to detect inconsistency (Lu and Ades, 2006; Dias *et al*., 2010; Lu *et al*., 2011; Higgins *et al*., 2012; Dias *et al*., 2013b), and they can be subdivided into three classes according to their approach to inconsistency. The ‘null’ approach, consisting only of the unrelated mean effects model, does not attempt to model inconsistency at all; it simply estimates each pair‐wise comparison separately. Inconsistency is then assessed by comparing the model fit and between‐study variance (heterogeneity) estimate of the pair‐wise comparisons against the results of the consistency model (Dias *et al*., 2013b). The ‘loop inconsistency’ approach proposes that inconsistency can only occur in closed loops in the evidence network and is exemplified by the inconsistency factors (Lu and Ades, 2006) and node splitting (Dias *et al*., 2010) models. The potential for loop inconsistency was first recognised in relation to indirect treatment comparisons (Bucher *et al*., 1997). These models increase the power with which inconsistency can be detected by limiting the degrees of freedom in the model. However, the presence of multi‐arm trials introduces ambiguities in how these models should be specified, especially for the inconsistency factors model. The ‘design inconsistency’ approach addresses this concern by introducing the concept of design inconsistency, in which *ABC* trials can be inconsistent with *AB* trials (Higgins *et al*., 2012). Essentially, the design inconsistency approach allocates additional degrees of freedom to resolve the ambiguity of loop inconsistency models. We view both the design‐by‐treatment‐interaction model (Higgins *et al*., 2012) and the two‐stage linear inference model (Lu *et al*., 2011) as belonging to this approach. The design inconsistency models also enable a global test for inconsistency across the network (Higgins *et al*., 2012), but the loop inconsistency models do not. On the other hand, the interpretation of individual parameters of the design‐by‐treatment‐interaction model is not straightforward because, in any multiparameter model, the meaning of each parameter depends on what other parameters are in the model. Conceptually, design inconsistencies are also hard to grasp: why would three‐arm trials result in systematically different results from two‐arm or four‐arm trials? Why would the included treatments be a better predictor of inconsistency than any other design or population characteristic?

Therefore, although the design inconsistency approach offers advantages, specifically unambiguous model specification and the global test for inconsistency, there are also reasons to favour the loop inconsistency approach. These are the clearer conception of inconsistency occurring in loops and the easier interpretation of local inconsistencies. The node‐splitting approach is especially attractive because inconsistency is evaluated one comparison at a time by separating the direct evidence on that comparison from the network of indirect evidence. The discrepancy between the estimates of relative treatment effects from these two sets of trials indicates the level of (in)consistency. However, node‐splitting analyses can be labour‐intensive, because each comparison of interest requires a separate model. Moreover, the analyst must decide which comparisons should be investigated, which is not trivial in the presence of multi‐arm trials. Finally, there may be several possible node‐splitting models for one comparison when it has been included in multi‐arm trials. In this paper, we present a decision rule to determine which comparisons to split that also ensures that each of the alternative node‐splitting models is valid. We build upon previous work on automated model generation for network meta‐analysis (van Valkenhoef *et al*., 2012a) to automatically generate the node‐splitting models.

Automation is not a substitute for proper understanding of the implemented statistical methods and their limitations. Rather, it reduces the effort that well‐versed users of the methods must expend, enabling them to focus on other issues. In addition, the statistical analysis of inconsistency is not a substitute for a thoughtful selection of trials prior to applying evidence synthesis. It is also unwise to investigate inconsistency alone while ignoring heterogeneity, as the two are closely related, and in one model, the heterogeneity parameter may absorb some of the variance that another model would classify as inconsistency. Finally, when significant inconsistency or excess heterogeneity is detected, the analyst faces the difficult question of how to address it. A careful analysis of the included trials and (local) discrepancies between their effect estimates is required to identify potential confounding factors. If a satisfactory explanation is found, the synthesis may be repaired, either by excluding the offending subset of trials or by correcting for the confounder through a meta‐regression analysis. Unexplained inconsistency or heterogeneity may mean that the meta‐analysis must be abandoned altogether, or at the very least must be interpreted with extreme caution.

## 2 Background

In this paper, we consider network meta‐analysis in a Bayesian framework (Dias *et al*., 2013a) and limit the discussion to homogeneous‐variance random‐effects models (Lu and Ades, 2004). First, we briefly review the consistency model, which is a simple extension of the Bayesian formulation of pair‐wise meta‐analysis. Then, we introduce node‐splitting models and, finally, review previous work on automated model generation for network meta‐analysis.

### 2.1 Consistency models

A network of evidence consists of a set of studies *S* numbered 1, …, *n*, where each study *S*_*i*_ has a number of arms that evaluate a set of treatments *T*(*S*_*i*_), where we assume that each arm evaluates a unique treatment (thus we may identify an arm by its treatment). Moreover, we assume that the studies form a connected network, that is, that there is a path between any two treatments included in the network.

Because the specific type of data and likelihood function are not important for the discussion that follows, we simply assume that for each treatment *x* ∈ *T*(*S*_*i*_)_,_ there is a parameter *θ*_*i*,*x*_ that expresses the effect of treatment *x* in study *S*_*i*_ on a linear additive scale. Thus, there is a likelihood of the form:

$${\text{data}}_{i}∼f_{i}\left(\theta_{i},\ldots \right)\text{,}$$

where ***θ***_*i*_ is the vector of treatment effects *θ*_*i*,*x*_. Then, for each study, we choose a reference treatment *b*(*i*) and express the treatment effects as:

$$\begin{array}{ll} \theta_{i,b\left(i\right)}=\mu_{i} & \\ \theta_{i,x}=\mu_{i}+\delta_{i,b\left(i\right),x} & x\neq b\left(i\right) \end{array}$$

Here, *μ*_*i*_ is the study‐specific effect of the reference treatment *b*(*i*) and *δ*_*i*,*b*(*i*),*x*_ is the *random effect* of *x* when compared with *b*(*i*) in study *S*_*i*_. Now

$$\delta_{i,b\left(i\right),x}∼N\left(d_{b\left(i\right),x},\sigma_{b\left(i\right),x}^{2}\right)\text{,}$$

where *d*_*b*(*i*),*x*_ is the *relative effect* of *x* compared with *b*(*i*), the quantity of interest, and $\sigma_{b\left(i\right),x}^{2}$ is the *random‐effects variance*, a measure of the heterogeneity between trials. In a *homogeneous‐variance* model, these variances are identical, $\sigma_{w,x}^{2}=\sigma_{y,z}^{2}=\sigma^{2}$, for all comparisons in the treatment network (*w*, *x*, *y*, *z* are treatments, and *w* ≠ *x*, *y* ≠ *z*). In such a model, the covariances between comparisons in multiarm trials work out to *σ* ^2^/2 (Higgins and Whitehead, 1996):

(1) $$\left(\begin{array}{c} \delta_{i,b\left(i\right),x}\\ \vdots \\ \delta_{i,b\left(i\right),z} \end{array}\right)∼N\left(\left(\begin{array}{c} d_{b\left(i\right),x}\\ \vdots \\ d_{b\left(i\right),z} \end{array}\right),\left(\begin{array}{ccc} \sigma^{2} & \sigma^{2}/2 & \cdots \\ \sigma^{2}/2 & \ddots & \sigma^{2}/2\\ \cdots & \sigma^{2}/2 & \sigma^{2} \end{array}\right)\right)$$

To complete the model, the exchangeability assumption renders the comparisons *consistent* (Lu and Ades, 2009): if we compare *x* and *y* indirectly through *z*, the result will be consistent with the direct comparison,

(2) $$d_{x,y}=d_{z,y}-d_{z,x}\text{.}$$

The right‐hand‐side parameters are the *basic parameters*, for which we estimate probability distributions. Although a network containing *m* treatments can have up to *m*(*m* − 1)/2 comparisons, it will have only *m−1* basic parameters. Any other relative effect can be calculated from the consistency relations. Hence *d*_*x*,*y*_, a *functional parameter*, is completely defined in terms of the basic parameters on the right‐hand side. Although the basic parameters are usually expressed relative to a common reference treatment (e.g. *z* in the aforementioned example), that is not a requirement (van Valkenhoef *et al*., 2012a).

### 2.2 Node‐splitting models

A node‐splitting analysis (Dias *et al*., 2010) splits one of the treatment comparisons, say *d*_*x*,*y*_, into a parameter for direct evidence $d_{x,y}^{dir}$ and a parameter for indirect evidence $d_{x,y}^{ind}$, in order to assess whether they are in agreement (i.e. the hypothesis is that $d_{x,y}^{dir}=d_{x,y}^{ind}$). The term node‐splitting may be confusing for some, because the treatment network represents a comparison as an edge rather than a node (or vertex). However, in the Bayesian hierarchical model, each parameter is represented by a node in a directed acyclic graph. When one of these parameters is split into two to evaluate conflict, the term node‐splitting is used. A node‐splitting analysis is thus performed separately for each of the comparisons in the treatment network on which both direct and indirect evidence are available, to assess evidence consistency.

Node‐splitting models are very similar to consistency models, except that the direct evidence for *d*_*x*,*y*_ is used alone to estimate $d_{x,y}^{dir}$, and a network meta‐analysis of the remaining evidence is used to estimate $d_{x,y}^{ind}$. The heterogeneity parameter *σ* ^2^ is shared between direct and indirect evidence to enable estimation even when the direct evidence consists of few trials. However, node‐splitting models for various comparisons and the consistency model will result in different estimates for *σ* ^2^, and comparing these estimates may also shed some light on potential inconsistencies (Dias *et al*., 2010). A two‐arm trial comparing *x* and *y* could thus be parameterised relative to the reference treatment *x* as:

$$\delta_{i,x,y}∼N\left(d_{x,y}^{dir},\sigma^{2}\right)\text{.}$$

**Figure 1:**
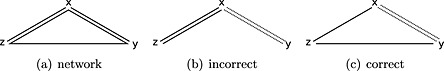
Evidence structure that requires a specific choice of reference treatments if we split *d*_*x*,*y*_. In (a), the evidence network is shown with lines to represent two‐arm trials and triangles to represent three‐arm trials. In (b) and (c), two possible parameterisations of the indirect evidence when the *xy* comparison is split are shown as solid lines: in (b), *x* is the reference treatment for the multi‐arm trial, and in (c), *y* is. The direct evidence is shown as dotted lines.

A four‐arm trial of *w*, *x*, *y* and *z*, with *x* as the reference treatment, would be parameterised as follows:

$$\left(\begin{array}{c} \delta_{i,x,y}\\ \delta_{i,x,w}\\ \delta_{i,x,z} \end{array}\right)∼N\left(\left(\begin{array}{c} d_{x,y}^{dir}\\ d_{x,w}\\ d_{x,z} \end{array}\right),\left(\begin{array}{ccc} \sigma^{2} & 0 & 0\\ 0 & \sigma^{2} & \sigma^{2}/2\\ 0 & \sigma^{2}/2 & \sigma^{2} \end{array}\right)\right)\text{,}$$

which generalises in a straightforward manner to any multi‐arm trials. Importantly, we do not want $d_{x,y}^{dir}$ to interact with any of the other d_*,*_, and thus, *δ*_*i*,*x*,*y*_ is given a distribution independent from the other relative effects in the study. If *d*_*x*,*y*_ has been investigated in multi‐arm trials, the node‐split model can be parameterised in multiple ways. In the aforementioned parameterisation of the *wxyz* trial, *x* has been chosen as the reference treatment, thus leaving the *y* arm of this trial out of the network of indirect evidence. We could alternatively have chosen *y* as the reference treatment, giving another (non‐equivalent) node‐splitting model, where the *x* arm is omitted from the indirect evidence. Figure 1 illustrates this for a three‐arm trial *xyz*: because there is no other evidence on the *yz* comparison, choosing *x* as the reference treatment for the multi‐arm trial results in a model in which there is no indirect estimate for *yz* (Figure 1(b)). This can be rectified by choosing *y* as the reference treatment instead (Figure 1(c)). Even if the model results in an indirect estimate with either choice of reference treatment, the choice of reference treatment may affect the results. These issues are discussed further in Section 3.3.

Only those comparisons where an indirect estimate can be made should be split, so if a comparison is not part of any loop in the evidence graph, it should not be considered. Multi‐arm trials complicate this situation somewhat, because evidence within a multi‐arm trial is consistent by definition. Thus, if we consider a situation where the evidence structure consists of only multi‐arm trials including *x*, *y* and *z*, then even though the comparison *d*_*x*,*y*_ is part of a loop, it cannot be inconsistent, and hence, no comparisons should be split. In complex networks that contain both two‐arm and multi‐arm trials, it may not be obvious whether there is potential inconsistency.

### 2.3 Note on relative‐effect data

In the earlier discussion, we assumed that the study data are available as absolute effects for each arm. However, data are often reported as contrasts, such as odds ratios or mean differences. Then, if the scale on which the relative effects were reported is compatible with the model, the likelihood becomes:

$${\text{data}}_{i}∼N\left(\delta_{i}^{\prime },\Sigma \right)\text{.}$$

where $\delta_{i}^{\prime }$ is the vector of contrasts reported for study *i*, typically expressed against a specific chosen reference treatment, which may differ from the desired reference treatment. The variance–covariance matrix Σ is fully determined by the marginal variances of each contrast and the variance of the absolute effect in the reference arm (Franchini *et al*., 2012). If ***δ***_*i*_ is the vector of relative effects against the desired reference treatment, then there is a matrix *A* such that ***δ***_*i*_^'^ = *A* ***δ***_*i*_. The likelihood then becomes:

$$data_{i}∼N\left(A\delta_{i},\Sigma \right)\text{.}$$

### 2.4 Automated model generation

Automated model generation for network meta‐analysis consists of generating the model structure (choosing the basic parameters and study reference treatments) and choosing appropriate priors and starting values (van Valkenhoef *et al*., 2012a). It was previously shown that for consistency models, the choice of basic parameters and study reference treatments is arbitrary, so long as the basic parameters form a spanning tree of the evidence network (van Valkenhoef *et al*., 2012a), but for inconsistency models that does not hold (Lu and Ades, 2006; van Valkenhoef *et al*., 2012b). A spanning tree is a sub‐network that connects all vertices of the original network, but contains no loops. To the best of our knowledge, no work has been published on model generation for node‐splitting models. General strategies for choosing vague priors and for generating starting values for the Markov chains are given in van Valkenhoef *et al*. (2012a).

The choice of prior for the heterogeneity parameter can have a large impact on its estimation, especially when few studies are available (Lambert *et al*., 2005). Because heterogeneity and inconsistency are closely linked, this choice will also affect the estimated degree of inconsistency. A similar phenomenon occurs in the frequentist framework, where the choice of estimators was shown to affect the detection of inconsistency (Veroniki *et al*., 2013). A sensitivity analysis may be necessary in some cases. An alternative or complementary approach is the use of prior data rather than heuristics or expert judgement to define the priors. A recent review of meta‐analyses published in the Cochrane library investigated the random‐effects variance commonly encountered in practice and stratified by outcome type, intervention type and medical specialty (Turner *et al*., 2012). The predictive distributions derived in that paper can be used as informative priors for the variance parameter *σ* ^2^ (Turner *et al*., 2012). A similar study provides informative priors for the variance in meta‐analyses on the standardised mean‐difference scale (Rhodes *et al*., 2015) and gives some guidance on how they may be applied on the mean‐difference scale as well. In principle, the same approach applies to other scales, and future research may produce the necessary data and methods.

## 3 Model generation

In a node‐splitting analysis of inconsistency, the first problem is deciding which comparisons can and should be assessed using a node‐splitting model. Then, given a comparison to be split, the usual model‐generation problems have to be solved. Priors and starting values for node‐splitting models can be chosen in the way described for consistency models (van Valkenhoef *et al*., 2012a), but generating the model structure may have some problems. If the comparison being split has only been assessed in two‐arm trials, the network of evidence for $d_{x,y}^{ind}$ can be analysed using a standard consistency model, and because the *xy* comparison must occur in a loop, the network is connected. Thus, as for consistency models, the choice of basic parameters and study reference treatments is arbitrary (van Valkenhoef *et al*., 2012a). However, in the presence of multi‐arm trials, more than just the comparison of interest may be removed from the network. As an example, the evidence network in Figure 1(a) has trials *xy*, *xz* and *xyz*. If we split *d*_*x*,*y*_ and choose *x* as the reference treatment for the *xyz* trial, $d_{x,y}^{ind}$ cannot be estimated (Figure 1(b)). This happens because the estimation of $d_{x,y}^{ind}$ requires an estimate of *d*_*y*,*z*_, but the *xyz* trial has been parameterised using *xy* and *xz*, so there is no remaining evidence on *yz*. If we choose *y* as the reference treatment, the problem disappears (Figure 1(c)). This problem was pointed out earlier for loop inconsistency models (Lu and Ades, 2006). Our strategy carefully chooses the comparisons to split so that such problems do not occur and that the choice of basic parameters and study reference treatments is again arbitrary.

### 3.1 Defining potential inconsistency

**Figure 2:**
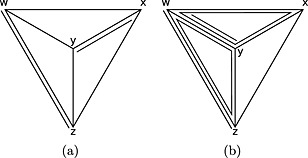
These evidence structures illustrate networks in which defining potential inconsistency is not straightforward. Two‐arm trials are shown as lines that stop short of vertices, three‐arm trials as triangles and four‐arm trials as tetrahedrons. (a) A network with one four‐arm and two two‐arm trials, where it is unclear whether loop inconsistency can occur. (b) A more complex network with one four‐arm, two three‐arm, and two two‐arm trials where the dependencies between potential loop inconsistencies are difficult to work out.

To arrive at a rule on whether to split specific comparisons, we require a definition of when a loop in the evidence network is (potentially) inconsistent. Because there is no clear‐cut distinction between inconsistency and heterogeneity (Higgins *et al*., 2012; Jansen and Naci, 2013), finding the right definitions is difficult. For example, in a network where three treatments (*x*, *y*, *z*) have been investigated in a three‐arm trial *xyz*, but only two out of three comparisons have been investigated in two‐arm trials (Figure 1(a)), it is unclear whether loop inconsistency could occur. Clearly, the two‐arm trials on *xy* and *xz* could disagree with the three‐arm trial; but if they do, this would manifest not only as a loop inconsistency, but also as heterogeneity on *xy* and *xz*. On the other hand, if the *x* arm had been omitted from the *xyz* trial, loop inconsistency could clearly be present. Our position is that investigating inconsistency of this loop could yield additional insight beyond looking at heterogeneity alone and thus that this should be carried out. The network in Figure 2(a) is similar, in that we could view the differences between the four‐arm trial *wxyz* and the two‐arm trials *wz* and *xy* as heterogeneity on those comparisons, or as loop inconsistency on the *wxyzw* loop or the *wyxzw* loop. However, unlike in the previous example, if we remove any of the arms of the four‐arm trial, no potentially inconsistent loops remain. Therefore, we consider any discrepancies between the two‐arm trials and the four‐arm trial in this network to be heterogeneity. To reiterate, because heterogeneity and inconsistency cannot always be distinguished, many of these distinctions are somewhat arbitrary and could have been made differently. For example, in the design‐by‐treatment‐interaction model, differences between two‐arm and three‐arm trials are considered to be ‘design inconsistencies’ (Higgins *et al*., 2012). Our definitions focus on loop inconsistency alone, as the node‐splitting model does not evaluate design inconsistency.

To determine whether a given loop is potentially inconsistent, we use the definition of Lu and Ades (2006): there must be at least three independent sources of evidence supporting the (three or more) comparisons in the loop. We define trials (i.e. sources of evidence) as independent if their treatment sets, *T*(*S*_*i*_), differ on treatments in the loop under consideration. For example, when judging whether the loop *xyzx* can be inconsistent, *wxy* and *xy* trials are considered the same because *w* does not occur in the loop. This is so because different estimates from studies that include the same set of treatments are more appropriately viewed as heterogeneity (Jansen and Naci, 2013). We adopt a stronger condition for longer loops: loops where two or more comparisons are included in exactly the same set of multi‐arm trials are not considered potentially inconsistent, because inconsistency occurring in such a loop can more parsimoniously be viewed as inconsistency in simpler loops, or as heterogeneity. By this definition, the network in Figure 1(a) contains a potentially inconsistent loop *xyzx*, because the comparison *xy* is supported by the *xy* and *xyz* studies, the *xz* comparison by the *xz* and *xyz* studies and the *yz* by the *xyz* study, and hence, no two comparisons are supported by exactly the same set of studies. Conversely, the network in Figure 2(a) does not contain a potentially inconsistent loop, because no matter how we construct the loop, at least two comparisons will be supported only by the four‐arm trial.

Our definition is in part motivated by the difficulties encountered by earlier work on loop inconsistency models. For example, in Figure 2(b), the *wxyzw* loop could be considered potentially inconsistent as it contains three independent sources of evidence, but it takes a longer path *wxy* through the three‐arm trial, which could be shortened to just *wy*, reducing the loop to *wyzw*. Doing so, however, would involve the two‐arm trial *wy*, which was not part of the original *wxyzw* loop. Not doing so, on the other hand, and considering the loops *wxyzw* and *wyzw* to be distinct inconsistencies, has the problem that these inconsistencies can only differ by heterogeneity on *wy* (van Valkenhoef *et al*., 2012b). Thus, although these (potential) inconsistencies are not strictly equal, treating them as different does not appear to be useful. It is difficult (and perhaps impossible) to precisely characterise these dependencies (van Valkenhoef *et al*., 2012b) because of the difficulty of distinguishing inconsistency from heterogeneity in the presence of multi‐arm trials. Fortunately, because node‐splitting models are relatively simple, we do not need to work out these dependencies explicitly. The stronger rule proposed earlier handles this gracefully: the loop *wxyzw* would not be considered potentially inconsistent because *wx* and *xy* are both contained in exactly the same set of studies, but the *wyzw* loop would be. Our definition of potential inconsistency of a loop can thus be summarised as the following two requirements:

- Among the comparisons in the loop, no two comparisons share the same set of supporting studies
- The loop has at least three comparisons, and no comparison or treatment occurs more than once

The formal graph‐theoretic definition is given in Appendix A.

### 3.2 Choosing the comparisons to split

We give a simple decision rule to determine whether to split a specific comparison, based on properties of the evidence structure that are easily verified:

For a given set of studies *S*, split *d*_*x*,*y*_ if and only if the modified network consisting of the studies *S*′ that do not include both *x* and *y* contains a path between *x* and *y*.

**Figure 3:**
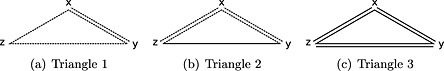
Some evidence structures and the nodes that will be split according to the proposed decision rule. Comparisons that will be split are shown as solid lines, and those that will not as dashed lines. Three‐arm trials are shown as triangles. In (a) no comparisons will be split, in (b) only the yz comparison will be split, and in (c) all three comparisons will be split.

Intuitively, *S*′ is the set of studies that could generate inconsistency on the *xy* comparison. An advantage of this approach is that we do not need to assess the global inconsistency degrees of freedom, which currently have no completely satisfactory definition and no efficient algorithm (Lu and Ades, 2006; van Valkenhoef *et al*., 2012b). Figure 3 shows a number of examples to demonstrate how the rule works. Figure 3(a) shows a structure in which no inconsistency can occur: disagreement between the two‐arm and three‐arm trials would be modelled as heterogeneity on *xy*. When we evaluate the rule for the *xy* comparison, the modified network is empty (contains no studies), and thus, we do not split *xy*. For the *xz* comparison, the modified network contains only the *xy* studies, so *x* is not connected to *z*, and we do not split *xz*. The *yz* comparison is similar to the *xy* comparison. By contrast, Figure 3(b) has three independent sources of evidence and thus is potentially inconsistent. Here, the rule selects only the *yz* comparison to split, as the reduced network consists of *xy* and *xz* studies, and thus, *y* and *z* are connected in the modified network. In theory, we could split all three comparisons, but *yz* is the only comparison in which the choice between including either of the other comparisons from the three‐arm trial in the indirect evidence network is arbitrary (also see Figure 1). In Figure 3(c), all comparisons have pair‐wise evidence, and thus, all comparisons are selected to be split. From the loop inconsistency perspective, splitting all three comparisons is redundant, yet using a node‐splitting model, each of the three will have different results. This is due to the way multi‐arm trials are handled, and, for each comparison, a different choice of reference treatment for the multi‐arm trial may also result in different results. However, because heterogeneity and inconsistency are so closely related, if inconsistency is less present in one of these models, heterogeneity would be greater. Therefore, it is important to consider both together.

In Appendix A, we prove that the decision rule corresponds to the definitions of potential inconsistency set out in Section 3.1. In particular, we show that in any potentially inconsistent loop, we split at least one comparison, and conversely, that any comparison selected to be split is part of a potentially inconsistent loop.

### 3.3 Implications for model generation

In Section 2.4, we remarked that when the network consists only of two‐arm trials, the model generation problem for a node‐splitting model can be decomposed into generating a model for a pair‐wise meta‐analysis of the direct evidence and generating a consistency model for the indirect evidence. However, in general, this does not hold for networks with multi‐arm trials (Figure 1). Fortunately, we show in this section that the model generation does decompose in this way if the comparisons to be split are chosen according to the decision rule proposed in the previous section.

First, if we split *xy*, the two usual parameterisations of a multi‐arm trial *S*_*i*_ can be summarised as follows:

1. Include the arms *T*(*S*_*i*_) − {*x*} in the model for $d_{x,y}^{ind}$.
2. Include the arms *T*(*S*_*i*_) − {*y*} in the model for $d_{x,y}^{ind}$.

A third parameterisation is also possible:

Include the arms *T*(*S*_*i*_) − {*x*, *y*} in the model for $d_{x,y}^{ind}$.

However, removing an additional arm from multi‐arm trials potentially decreases the precision of the indirect estimate $d_{x,y}^{ind}$. If we decide to include either the *x* arm or the *y* arm of multi‐arm trials, we can either consistently include the same arm for all trials – pure option (1) or (2), or make this decision individually for each trial – a mixture of options (1) and (2). In the pure case, there are two alternative models with potentially different results, whereas in the mixture case, there are 2^*k*^ alternative models, where *k* is the number of multi‐arm trials that include both *x* and *y*.

**Figure 4:**
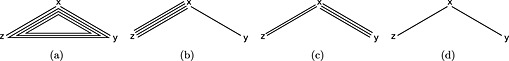
When splitting the *yz* comparison of the network shown in (a), the indirect evidence can be parameterised in 2^3^ + 1 = 9 ways owing to the three three‐arm trials that include *yz*. Three such ways are shown here: (b) consistently include the *xz* comparison of the three‐arm trials; (c) include *xy* for some trials and *xz* for others; (d) include neither the *xy* nor the *xz* comparison.

This is illustrated for a simple network in Figure 4. Usually, only the pure options are considered (Dias *et al*., 2010), but one could argue that choosing a different included treatment in different trials can result in a more balanced evidence network, and might thus be preferred. In any case, exploring all 2^*k*^ + 1 alternative models is generally infeasible, which is probably why it has not received any attention. Moreover, given the computationally intensive nature of model estimation, even estimating the two alternative models that correspond to options (1) and (2) is undesirable, and in practice, one of them is chosen arbitrarily.

Now we show that model generation is trivial if the comparisons to be split are chosen according to the decision rule and if we parameterise the node‐splitting model according to option (3), and by extension that it is also trivial if we use option (1) or (2) instead. First, if the reduced network defined by the decision rule contains components that are not connected to the comparison *xy* under consideration, then we can safely remove those components from the network because they will not contribute to inconsistency on *xy*. However, it may be desirable to include disconnected components in the model to estimate the heterogeneity parameter, especially if estimates of this parameter are being compared between models. In addition, the decision rule guarantees that *x* and *y* are connected even in the absence of any trials that include the *xy* comparison. Given this, the network of indirect evidence can simply be analysed using a consistency model that connects *x* and *y* indirectly, so its parameterisation is arbitrary, and existing algorithms can be applied (van Valkenhoef *et al*., 2012a). Any disconnected components can be parameterised similarly and estimated in a single model in which the heterogeneity parameter is shared. Moreover, the direct evidence can be synthesised in a pair‐wise model, which is also trivial to parameterise.

This discussion extends to options (1) and (2) because *x* and *y* are already connected in the network of indirect evidence, so adding one of these arms back into the relevant multi‐arm trials will again result in a connected network, which can be parameterised as a consistency model with the amendment that the study reference effect parameter *μ*_*i*_ will be shared between the two sub‐models. The model corresponding to option (3) has no such shared reference treatment, as each multi‐arm study that includes the comparison being split is subdivided into two virtual studies: one including the two treatments of interest and another containing all remaining arms. If the second virtual study contains only a single arm, it can be eliminated altogether because it provides no information on relative effects.

Thus, however we decide to parameterise the node‐splitting model, generating the model is trivial if the comparison being split was chosen according to the decision rule proposed in the previous section. The 2^*k*^ + 1 alternative parameterisations correspond to 2^*k*^ mixtures of options (1) and (2) and a single model corresponding to option (3) described earlier. If a single model is to be estimated, one could argue that one of the 2^*k*^ mixtures of options (1) and (2) is preferred because these models make fuller use of the evidence, or that option (3) should be preferred because it results in a unique model that more closely mimics a consistency model.

## 4 Implementation and example

The methods have been implemented in version 0.6‐1 of the gemtc package (http://cran.r-project.org/package=gemtc) for the R statistical software (http://www.r-project.org). Source code is available on GitHub: https://github.com/gertvv/gemtc/tree/0.6‐1. gemtc currently generates node‐splitting models according to option (3): for multi‐arm trials that include the comparison being split, it includes neither treatment of that comparison in the network of indirect evidence. If the evidence network becomes disconnected as a result, the disconnected components are not discarded, but are included in the model to aid the estimation of the heterogeneity parameter. The R package can generate and estimate all relevant node‐splitting models according to the decision rule proposed in this paper and summarise the results textually or graphically. Estimation uses standard Markov chain Monte Carlo software, and the package requires one of JAGS (Plummer, 2003), OpenBUGS (Lunn *et al*., 2009), or WinBUGS (Lunn *et al*., 2000), to be installed, as well as the corresponding R package. Because it is more actively maintained and integrates more nicely with R, we recommend JAGS and the rjags package.

**Figure 5:**
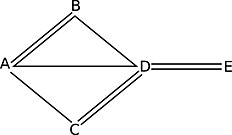
Evidence network for the Parkinson's disease dataset. A = placebo; B = pramipexole; C = ropinirole; D = bromocriptine; E = cabergoline.

In this section, we illustrate the methods and implementation using a worked example based on a real‐life evidence network. The dataset consists of seven trials comparing placebo against four dopamine agonists (pramipexole, ropinirole, bromocriptine and cabergoline) as adjunct therapy for Parkinson's disease (Franchini *et al*., 2012). Parkinsons patients often experience fluctuations in their response to treatment: ‘on‐time’ periods when the drugs appear to be effective alternate with ‘off‐time’ periods when symptoms are not under control. We compare the drugs' ability to reduce the amount of ‘off‐time’ relative to the amount of ‘off‐time’ on placebo (both in conjunction with the background therapy). The data are summarised in Table 1, and the treatment network is shown in Figure 5. Naturally, automation is most useful for large and complex networks, but a small network makes the example easier to follow.

**Table 1:** Mean off‐time reduction (hours) data from seven trials studying treatments for Parkinson's disease (Franchini *et al*., 2012).

Study	Treatment	Mean	Standard deviation	Sample size
1	A	− 1.22	3.70	54
	C	− 1.53	4.28	95
2	A	− 0.70	3.70	172
	B	− 2.40	3.40	173
3	A	− 0.30	4.40	76
	B	− 2.60	4.30	71
	D	− 1.20	4.30	81
4	C	− 0.24	3.00	128
	D	− 0.59	3.00	72
5	C	− 0.73	3.00	80
	D	− 0.18	3.00	46
6	D	− 2.20	2.31	137
	E	− 2.50	2.18	131
7	D	− 1.80	2.48	154
	E	− 2.10	2.99	143

A = placebo; B = pramipexole; C = ropinirole; D = bromocriptine; E = cabergoline.

The Parkinson dataset is included with the gemtc package, so we can load it as follows:

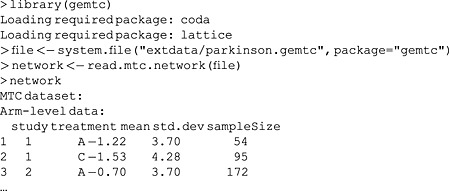

Here, lines that start with a ‘>’ signify commands entered into R, and lines that do not are output of those commands. The output has been truncated (indicated by ‘…’) for inclusion in the paper, and R will display the full dataset given in Table 1. As aforementioned, we use system.file to find an XML file included with the gemtc package (produced using the discontinued Java‐based GeMTC graphical user interface) and load it using read.mtc.network. For new datasets, it is more convenient to use mtc.network to construct networks from R data frames structured like the previous output. In addition, mtc.data.studyrow can convert the one‐study‐per‐row format commonly used in BUGS code to the format used by gemtc. The package has a wide range of features for working with network meta‐analysis datasets and models, such as evidence network plots, convergence assessment diagnostics and plots and output summaries and visualisations. In this section, we only present the specific functionality for node‐splitting, and we refer the interested reader to the manual of the gemtc package for further information.

Using the mtc.nodesplit.comparisons command, we can see which comparisons the decision rule elects to split for this network (Figure 5):

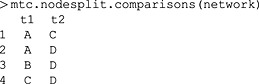

The decision rule selects the *AC*, *AD*, *BD* and *CD* comparisons, but not *AB* or *DE*. *AC* and *CD* are selected because they only occur in two‐arm trials and are clearly still connected if those trials are removed from the network. Conversely, the *DE* comparison clearly has no indirect evidence.

The three comparisons involving the three‐arm trial are more interesting. The *AD* comparison is selected because, if we remove the three‐arm trial from the network, *AD* is still connected through the *AC* and *CD* trials. Similarly, the *BD* comparison remains connected through *BA*, *AC* and *CD* trials. Finally, the *AB* comparison is not split because, if the *ABD* and *AB* trials are removed from the network, there is no longer a connection between *A* and *B*. It could be argued that splitting only one of the *AC*, *BD* or *CD* comparisons might be sufficient to investigate inconsistency in the *ACDBA* loop. However, as we pointed out earlier, such dependencies are difficult to work out for more complex networks, and we accept potential redundant testing such as this to be able to test for inconsistency wherever in the network it may reasonably exist.

To automatically run node‐splitting models for all of the comparisons selected by the decision rule, we can use the mtc.nodesplit function. This function accepts a number of arguments to modify which comparisons it will split as well as the priors, starting values and number of iterations, for which we again refer to the gemtc manual.

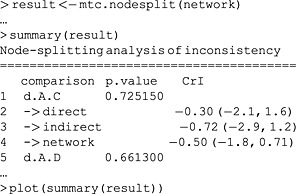

**Figure 6:**
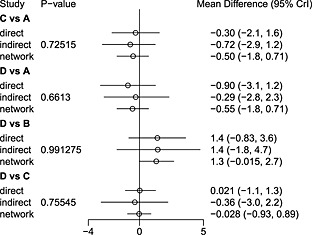
Summary of a node‐splitting analysis consisting of four separate node‐splitting models and a consistency model. A = placebo; B = pramipexole; C = ropinirole; D = bromocriptine; E = cabergoline.

The output of the plot command in Figure 6 visually conveys the information in the summary (truncated in the output). In this case, it would appear that the results from direct and indirect evidence are in agreement with each other and with the results of the consistency model. This is also reflected by the inconsistency *P*‐values, which are far from concerning. Because of the small number of included trials, and consequently low power to detect differences, this is not too surprising.

It is also possible to more closely inspect the results of individual models. For example, to inspect heterogeneity statistics:

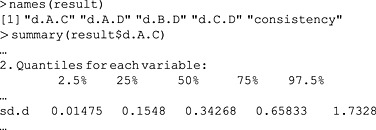

The summary is typically quite long, giving both moments and quantiles for all parameters in the model, and the output is heavily truncated to highlight the between‐studies standard deviation. The following code computes the median between‐studies standard deviation for all five models:

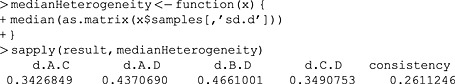

In this case, the estimated heterogeneity in each of the node‐splitting models is larger than in the consistency model because the node‐splitting model has more degrees of freedom, resulting in reduced power to estimate the heterogeneity parameter. If it were smaller in the node‐splitting models, this would indicate that splitting that comparison explained some of the heterogeneity observed in the consistency model and thus that there is reason to suspect inconsistency. It may also be useful to inspect the densities (Dias *et al*., 2010), which can be achieved as follows:

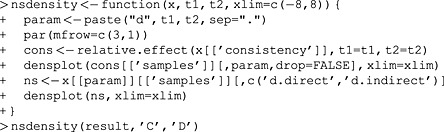

**Figure 7:**
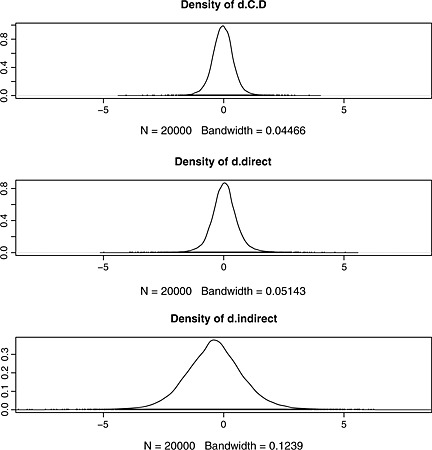
Comparison of posterior densities estimated for the *CD* comparison from the consistency model (top), and direct (middle) and indirect (bottom) evidence from the node‐splitting model. *N* is the sample size, and ‘Bandwidth’ is a parameter of the kernel density estimation that is used to produce smooth density plots. The coda package automatically sets the bandwidth as a function of the standard deviation, the interquartile range and the size of the sample.

Here, we first define a function that, given the results of a node‐splitting analysis, plots the densities relevant to a specific comparison in three rows using the densplot function from the coda package. Then we invoke it to produce a plot of the densities for the *CD* comparison, shown in Figure 7. Again, direct and indirect evidence appear to be in broad agreement, and the consistency‐model result is more precise than either the direct or the indirect evidence.

As for any analysis using Markov chain Monte Carlo techniques, it is important to assess convergence. The package supports a number of ways to do this for individual models, mostly provided by the coda package; for details, we refer to the documentation of gemtc and coda. Convergence was sufficient for each of the five models estimated in this analysis.

## 5 Conclusion

In this paper, we provide methods to automatically generate the models required for the assessment of inconsistency using node‐splitting models. Our work advances the state of the art in two ways. First, we provide an unambiguous decision rule for choosing the comparisons to be split and prove that it will select all comparisons of interest and only comparisons of interest. The decision rule improves upon the rule originally proposed (Dias *et al*., 2010) by being fully unambiguous, less computationally expensive and proven correct under a specific definition of potential inconsistency. Second, although each comparison to be split may allow several alternative parameterisations, we prove that for each comparison selected by the decision rule, generating the model is trivial. This is not true for every comparison that occurs in a potentially inconsistent loop; it required careful design of the decision rule.

Our methods have a number of limitations. First, although automation reduces the impact of some of the drawbacks of the node‐splitting approach, it does not eliminate them. Ambiguities still exist in which nodes to split and how to parameterise the model, and these may affect the results to some extent. A large number of models must still be run, and this process may be time‐consuming for larger networks. Second, especially in small networks, the decision rule tends to split more comparisons than there are potentially inconsistent loops. Future work could investigate methods for reducing such redundancies. However, it seems unlikely that redundancies can be eliminated completely, so such approaches are likely to also be heuristic.

Finally, the assessment of heterogeneity and inconsistency remains a challenge, especially because in many circumstances that involve multi‐arm trials, there is no clear distinction between the two. One model may detect an inconsistency, whereas another model detects high heterogeneity but no inconsistency. However, this situation is not problematic because the response in both cases should be the same: to investigate the cause of the observed inconsistency or heterogeneity. This holds whether it is a three‐arm trial that differs from a set of two‐arm trials, a two‐arm trial that differs from other two‐arm trials, or any other case. Hopefully, such an investigation will yield insight into the cause of heterogeneity or inconsistency, such as differences in population, study sponsorship or intervention definitions.

## Appendix A Proof of correctness of the decision rule

In the proofs, we use some standard notions from graph and set theory; in particular, we refer to loops as cycles and to comparisons as edges. We follow van Valkenhoef *et al*. (2012a) in defining the network (graph) of treatment comparisons, which we take to be undirected.
*(Potential inconsistency)*
Let each edge (i.e. comparison) be the set of the vertices (i.e. treatments) it connects: *e* = {*x*, *y*}. Denote the set of studies that include an edge *e* as *r*(*e*) = {*S*_*i*_ ∈ *S* : *e* ⊂ *T*(*S*_*i*_)}. Let a cycle (i.e. loop) then be the ordered list *C* = (*e*_1_, …, *e*_*n*_) of its edges *e*_*i*_, *i* ∈ {1, …, *n*}. A cycle *C* = (*e*_1_, …, *e*_*n*_) is potentially inconsistent if and only if *n* > 2 and each of its edges has a unique set of supporting studies: ∀ *i*, *j* : *r*(*e*_*i*_) = *r*(*e*_*j*_) ⇒ *i* = *j*.
Lemma 1 If a cycle is potentially inconsistent, at least one of its edges will be split.
Consider a potentially inconsistent cycle *C* = (*e*_1_, …, *e*_*n*_). According to the decision rule, an edge *e*_*i*_ = {*x*, *y*} will be split if $\forall j\neq i\;:\;r\left(e_{i}\right)⊅r\left(e_{j}\right)$, because only those edges that are included in some studies where *e*_*i*_ is not will survive the removal of the studies that include *e*_*i*_. We show by contradiction that such an edge must exist. Assume that there is no edge *e*_*i*_ such that $\forall j\neq i\;:\;r\left(e_{i}\right)⊅r\left(e_{j}\right)$. Then, for any edge *e*_*i*_, we can find another edge *e*_*j*_ such that *r*(*e*_*i*_) ⊃ *r*(*e*_*j*_). Further, *r*(*e*_*i*_) ≠ *r*(*e*_*j*_), so *r*(*e*_*i*_) ⊋ *r*(*e*_*j*_). By repeated application of this fact, we can construct a permutation *p*(*i*) of the edges such that *r*(*e*_*p*(1)_) ⊋ *r*(*e*_*p*(2)_) ⊋ … ⊋ *r*(*e*_*p*(*n*)_). However, there must then also be an edge *e*_*i*_ such that *r*(*e*_*p*(*n*)_) ⊋ *r*(*e*_*i*_), which contradicts the strict order we just constructed. □
Lemma 2 If an edge is split, it occurs in at least one potentially inconsistent cycle.
If the edge *e*_1_ = {*x*, *y*} is split, then it is part of at least one cycle *C* = (*e*_1_, …, *e*_*n*_), *n* > 2, where ∀ *i* > 1 : *r*(*e*_1_) ⊅ *r*(*e*_*i*_), and hence, ∀ *i* > 1 : *r*(*e*_1_) ≠ *r*(*e*_*i*_). Suppose *e*_*n*_ = {*w*, *x*} and *e*_2_ = {*y*, *z*}, then *r*(*e*_2_) must contain at least some studies that do not include *x* and *r*(*e*_*n*_) must contain some studies that do not include *y*. By definition, all studies in *r*(*e*_2_) include *y* and all studies in *r*(*e*_*n*_) include *x*. Thus, *r*(*e*_2_) ≠ *r*(*e*_*n*_), *r*(*e*_1_) ≠ *r*(*e*_2_), and *r*(*e*_1_) ≠ *r*(*e*_*n*_), so there are at least three distinct sets of supporting studies. Now, if for any *i*, *j* > 1, *i* < *j*, we have *r*(*e*_*i*_) = *r*(*e*_*j*_), and *e*_*i*_ = {*t*, *u*}, *e*_*j*_ = {v, *w*}, *t* ≠ *w*, then we can create a shorter cycle *C*′ = (*e*_1_, …, *e*_*i* − 1_, {*t*, *w*}, *e*_*j* + 1_, …, *e*_*n*_). *C*′ has length > 2 unless *r*(*e*_2_) = *r*(*e*_*n*_), which we already showed is not the case. Moreover, we have *r*({*t*, *w*}) ⊃ *r*(*e*_*i*_) = *r*(*e*_*j*_), so $r\left(e_{1}\right)⊅r\left(\left\{t,w\right\}\right)$. The cycle *C*′ has the same properties as *C*, so we can apply this step repeatedly to obtain a series *C*, *C*′, *C*″, … of cycles of successively smaller length. Finally, there must be a cycle *C** = (*e*_1_, …, *e*_*m*_), with *m* > 2, where ∀ *i*, *j* : *r*(*e*_*i*_) = *r*(*e*_*j*_) ⇒ *e*_*i*_ = *e*_*j*_. □
